# GnRH Antagonists Produce Differential Modulation of the Signaling Pathways Mediated by GnRH Receptors

**DOI:** 10.3390/ijms20225548

**Published:** 2019-11-07

**Authors:** Samantha Sperduti, Silvia Limoncella, Clara Lazzaretti, Elia Paradiso, Laura Riccetti, Sara Turchi, Ilaria Ferrigno, Jessika Bertacchini, Carla Palumbo, Francesco Potì, Salvatore Longobardi, Robert P. Millar, Manuela Simoni, Claire L. Newton, Livio Casarini

**Affiliations:** 1 Unit of Endocrinology, Department of Biomedical, Metabolic and Neural Sciences, University of Modena and Reggio Emilia, Via P. Giardini 1355, 41126 Modena, Italy; 2 Center for Genomic Research, University of Modena and Reggio Emilia, Via G. Campi 287, 41125 Modena, Italy; 3 International PhD School in Clinical and Experimental Medicine (CEM), University of Modena and Reggio Emilia, Via G. Campi 287, 41125 Modena, Italy; 4 Department of Biomedical, Metabolic Science and Neuroscience, University of Modena and Reggio Emilia, Via G. Campi 287, 41125 Modena, Italy; 5 Department of Medicine and Surgery, Unit of Neurosciences, University of Parma, Via Volturno 39/E, 43125 Parma, Italy; 6 Global Clinical Development, R and D Merck KGaA, Frankfurter Strasse 250, 64293 Darmstadt, Germany; 7 Centre for Neuroendocrinology and Department of Immunology, Faculty of Health Sciences, University of Pretoria, Lynnwood Road and Roper Street, P.O. Box 2034, Pretoria 0001, South Africa; 8 Unit of Endocrinology, Department of Medical Specialties, Azienda Ospedaliero-Universitaria, Via P. Giardini 1355, 41126 Modena, Italy; 9 Physiologie de la Reproduction et des Comportements (PRC), Institut National de la Recherche Agronomique (INRA), Centre National de la Recherche Scientifique (CNRS), Institut Français du Cheval et de l’Equitation (IFCE), Université de Tours, 37380 Nouzilly, France

**Keywords:** Gonadotropin-releasing hormone (GnRH), antagonist, pituitary, luteinizing hormone (LH), follicle-stimulating hormone (FSH), gonadotropins, assisted reproduction techniques (ART), Cetrorelix, Ganirelix, Teverelix

## Abstract

Commercial gonadotropin-releasing hormone (GnRH) antagonists differ by 1–2 amino acids and are used to inhibit gonadotropin production during assisted reproduction technologies (ART). In this study, potencies of three GnRH antagonists, Cetrorelix, Ganirelix and Teverelix, in inhibiting GnRH-mediated intracellular signaling, were compared in vitro. GnRH receptor (GnRHR)-transfected HEK293 and neuroblastoma-derived SH-SY5Y cell lines, as well as mouse pituitary LβT2 cells endogenously expressing the murine GnRHR, were treated with GnRH in the presence or absence of the antagonist. We evaluated intracellular calcium (Ca^2+^) and cAMP increases, cAMP-responsive element binding-protein (CREB) and extracellular-regulated kinase 1 and 2 (ERK1/2) phosphorylation, β-catenin activation and mouse luteinizing-hormone β-encoding gene (*Lhb*) transcription by bioluminescence resonance energy transfer (BRET), Western blotting, immunostaining and real-time PCR as appropriate. The kinetics of GnRH-induced Ca^2+^ rapid increase revealed dose-response accumulation with potency (EC50) of 23 nM in transfected HEK293 cells, transfected SH-SY5Y and LβT2 cells. Cetrorelix inhibited the 3 × EC_50_ GnRH-activated calcium signaling at concentrations of 1 nM–1 µM, demonstrating higher potency than Ganirelix and Teverelix, whose inhibitory doses fell within the 100 nM–1 µM range in both transfected HEK293 and SH-SY5Y cells in vitro. In transfected SH-SY5Y, Cetrorelix was also significantly more potent than other antagonists in reducing GnRH-mediated cAMP accumulation. All antagonists inhibited pERK1/2 and pCREB activation at similar doses, in LβT2 and transfected HEK293 cells treated with 100 nM GnRH. Although immunostainings suggested that Teverelix could be less effective than Cetrorelix and Ganirelix in inhibiting 1 µM GnRH-induced β-catenin activation, *Lhb* gene expression increase occurring upon LβT2 cell treatment by 1 µM GnRH was similarly inhibited by all antagonists. To conclude, this study has demonstrated Cetrorelix-, Ganirelix- and Teverelix-specific biased effects at the intracellular level, not affecting the efficacy of antagonists in inhibiting *Lhb* gene transcription.

## 1. Introduction

Gonadotropin releasing hormone (GnRH) is secreted by hypothalamic GnRH-expressing neurons and regulates mammalian reproductive functions. It is a decapeptide released in a pulsatile fashion into the hypophyseal portal blood system and acts on GnRH receptor (GnRHR)-expressing gonadotrope cells of the anterior pituitary, triggering the synthesis and secretion of the luteinizing (LH) and follicle-stimulating (FSH) hormones [1,2,3].

GnRHR is a G-protein coupled receptor (GPCR) [4,5], and its main effector in pituitary cells is the Gα_q/11_ protein, the activation of which results in phosphoinositide phospholipase Cβ (PLCβ) stimulation and subsequent production of inositol (1,4,5)-trisphosphate (IP_3_) and diacylglycerol (DAG) [6]. IP_3_ induces intracellular Ca^2+^ release by the endoplasmic reticulum, which is linked to gonadotropin secretion and activation of the calmodulin/calcineurin/NFAT- and calmodulin/ Ca^2+^ calmodulin-dependent protein kinase II (CaMKII)-pathway, as well as in further Ca^2+^ influx through L-type voltage gated calcium channels [2]. However, GnRHR modulates the simultaneous activation of multiple intracellular signaling cascades, depending on cell specific-contexts [7,8]. DAG mediates protein kinase C (PKC) activation and downstream phosphorylation of the extracellular signal-regulated kinase 1 and 2 (ERK1/2), jun-N-terminal kinase (JNK), and p38 mitogen-activated protein kinases (MAPKs) [2,9,10,11]. GnRHR coupling to other heterotrimeric G-protein subunits, such as the Gα_s_ [12,13,14] and Gα_i_ [15], has also been described [6,16]. Gα_s_ protein activation by GnRHR-hormone binding results in adenylyl cyclase and (cAMP) increase, and cAMP response element-binding protein (CREB) phosphorylation via protein kinase A (PKA) activation. cAMP production may also be induced by alternative pathways involving the Gα_q/11_ protein and specific PKC isoforms [17], implementing the complexity of GnRHR signaling signature. All of these intracellular events precede the expression of *LHB* and *FSHB* target genes [13,18]. Finally, another target of GnRH-mediated signal transduction is β-catenin activation [19,20]. β-catenin acts as a dual-function protein, participating in both cell-adhesion, as a member of the adherens junction, and in the regulation of *LHB* and Wnt-target gene transcription [21,22,23] after translocation into the cell nucleus [19,24].

**Figure 1:**
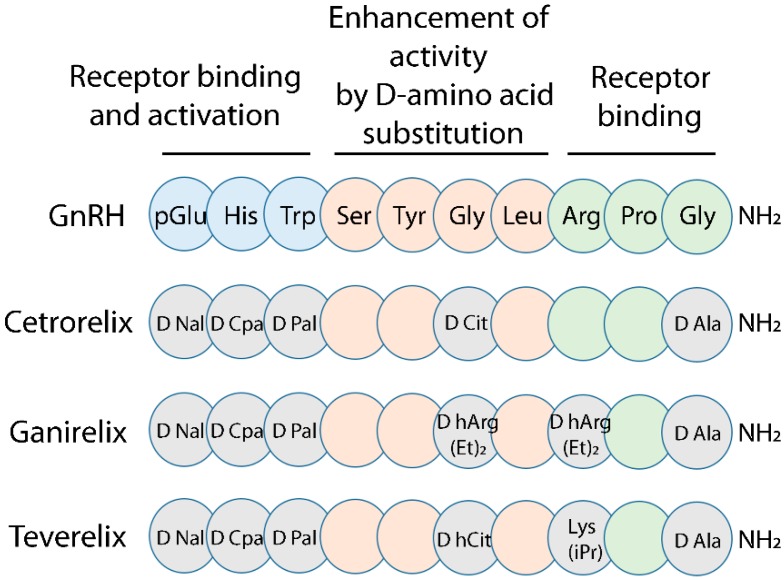
Amino acid sequence of mammalian gonadotropin releasing hormone (GnRH) and antagonists. Substitution of amino acids at position 6 (orange) by D-amino acids increases binding affinity and decreases metabolic clearance, resulting in increased activity of the compound. The COOH-terminal domain (Arg-Pro-Gly-NH_2_ group; green) is involved in receptor binding, while the NH_2_-terminal domain (pGlu-His-Trp; blue) is involved in both the receptor binding and activation. Amino acid substitutions falling within the C-terminal region produce antagonists and are indicated by the multiple letter code. The image is adapted from Millar et al. [5].

GnRH agonists and antagonists are useful to control gonadotropin production, in the context of assisted reproduction technologies (ART), as well as for the treatment of certain hormone-dependent diseases [25,26,27]. GnRH antagonists are typically decapeptides structurally similar to GnRH, differing from the native hormone by a few amino acids which results in reversible GnRHR binding without activation [5,28]. The GnRH antagonists Cetrorelix, Ganirelix and Teverelix, share highly similar structure (Figure 1), differing by only two amino acids at position 6 and 8 of the protein chain [5,26]. While the effects of these different GnRH antagonists have never been comprehensively compared in vitro, the use of Cetrorelix and Ganirelix to prevent premature ovulation is considered to lead to similar clinical outcomes [29,30], while Teverelix, although potentially useful for clinical purposes, has not yet been commercially marketed [31,32,33]. Although they share a high degree of similarity, the molecular differences between the antagonists lead to the hypothesis that antagonist-specific, biased effects on GnRHR-dependent pathways may occur upon receptor binding, resulting in ligand-induced selective signaling (LiSS) [34].

In cell lines expressing GnRHR, we compared Cetrorelix, Ganirelix and Teverelix in inhibiting a range of GnRH-induced intracellular signaling cascades, in vitro. This study improves the knowledge of the structure–function relationship of GnRH antagonists and provides results useful to develop drugs for personalized clinical applications.

## 2. Results

### 2.1. Gonadotropin Releasing Hormone (GnRH) Antagonist-Induced Inhibition of Intracellular Ca^2+^ Increase

In order to find the optimal GnRH dose to evaluate the action of antagonists in inhibiting the intracellular Ca^2+^ increase, dose-response experiments were performed. Thus, Ca^2+^ biosensor-expressing cells were treated by increasing concentrations of GnRH (pM–µM range) and luminescent signals corresponding to the intracellular Ca^2+^ concentration were measured by BRET. GnRH-mediated Ca^2+^ accumulation was measured in transiently transfected HEK293/GnRHR and SH-SY5Y/GnRHR cells, and in a LβT2 cell line, naturally expressing the murine GnRHR [35].

**Figure 2:**
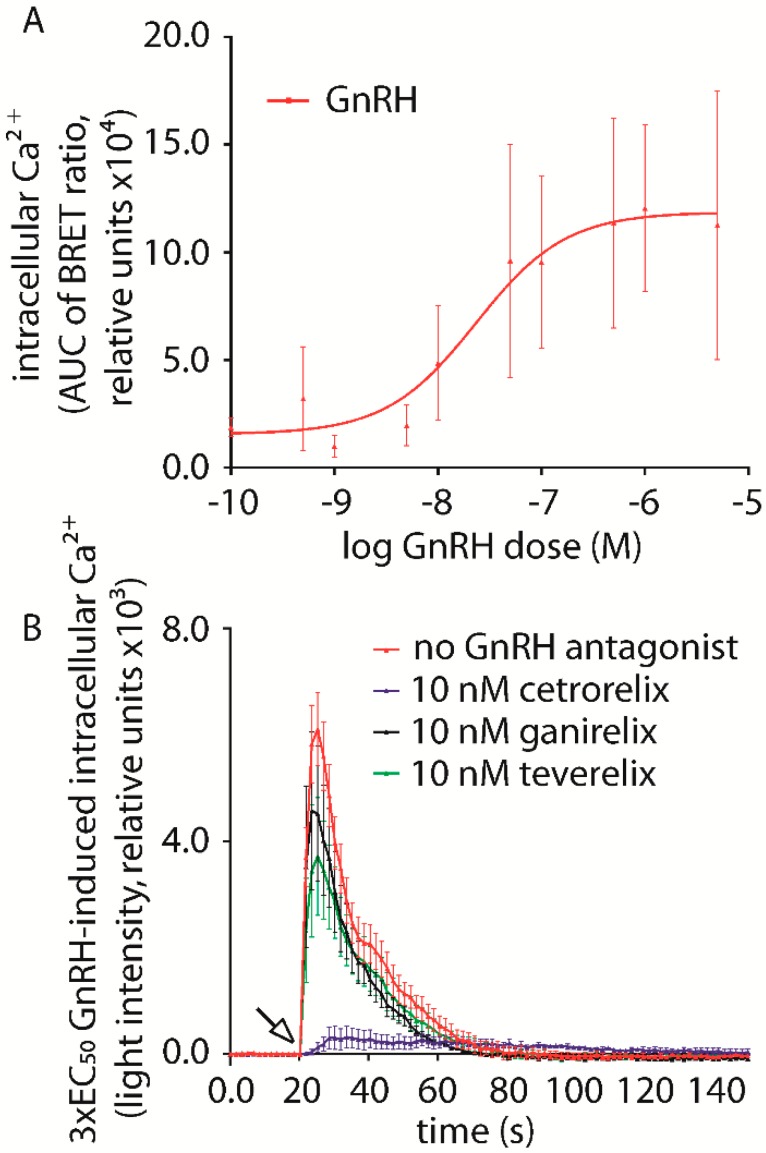
Analysis of the kinetics of GnRH-induced intracellular Ca^2+^ increase, in HEK293/GnRHR cells, in the presence or in the absence of GnRH and antagonists. (**A**) Ca^2+^ BRET biosensor signal was measured over 150 s and the arrow indicates the GnRH injection (pM–µM range) that occurred at the 20 s time-point. AUCs were calculated from kinetic data and represented as means ± SEM. The dose-response curve was obtained by interpolating means of AUC data using non-linear regression (EC_50_ = 23.26 ± 3.37 nM; means ± SEM; *n* = 3). (**B**) Kinetics of 3 × EC_50_ GnRH-induced intracellular Ca^2+^ increase, in the presence or in the absence of 10 nM Cetrorelix, Ganirelix and Teverelix. Light emissions are represented in the X-Y graph as means ± SEM and consecutive points were connected by lines. AUC of antagonists were calculated and statistically compared (Cetrorelix AUC = 21,482 ± 6718; Ganirelix AUC = 73,164 ± 16,237 *; Teverelix AUC = 74,321 ± 17,569 *; 3 × EC_50_ GnRH AUC = 109,340 ± 13,866 *; * = significantly different versus Cetrorelix AUC; Kruskal-Wallis test; *p* < 0.005; means ± SEM; *n* = 6).

Upon GnRH injection, intracellular Ca^2+^ rapidly increased, achieving the maximal level within about 5 s, before decreasing back to the basal level within about 80 s. No response was observed upon injection of vehicle (negative control). AUCs obtained from Ca^2+^ activation kinetics were plotted against the GnRH concentration in a X-Y graph. Data were interpolated by non-linear regression and the potency (EC_50_) of GnRH in inducing the intracellular ion increase in HEK293/GnRHR cells was calculated to be 23.26 ± 3.37 nM (Figure 2A). GnRH-induced intracellular Ca^2+^ accumulation was also observed in both the SH-SY5Y/GnRHR and LβT2 cell lines (SH-SY5Y/GnRHR EC_50_ = 5.78 ± 3.04 nM; LβT2 EC_50_ = 1.80 ± 2.88 nM; Supplementary Figure S1). For all cell lines, GnRH potency was similar and fell within the nM range (Kruskal-Wallis test; *p* ≥ 0.05; n = 3).

Potencies of Cetrorelix, Ganirelix and Teverelix in inhibiting the hormone-induced intracellular Ca^2+^ increase were then compared in vitro. Each of the cell lines were treated by a fixed concentration of GnRH corresponding to the three-fold higher dose than the calculated EC_50_ (3 × EC_50_; a concentration optimized for evaluating signal variations in inhibition experiments), in the presence or absence of increasing antagonist (Cetrorelix, Ganirelix or Teverelix) concentrations. Data representing the kinetics of intracellular Ca^2+^ increase, evaluated over 150 s by BRET were compared after AUC calculation.

Analysis revealed that the antagonists have different potencies in inhibiting the GnRH-induced intracellular Ca^2+^ increase (Figure 2B). In HEK293/GnRHR cells, the highest inhibition of the GnRH-induced signal was observed with 10 nM Cetrorelix, with an area under the curve (AUC) of 21,482 ± 6718. The same concentration (10 nM) of Ganirelix and Teverelix resulted in different inhibitory effects. For Ganirelix, the AUC was 73,164 ± 16,237 while for Teverelix it was 74,321 ± 17,569. The AUC for HEK293/GnRHR cells without GnRH antagonist treatment was 109,340 ± 13,866. When compared with the inhibition induced by Cetrorelix, significant differences were observed for all treatments (*p* = 0.005) (Figure 2B). These results were confirmed after comparing the AUCs obtained with a number of antagonist concentrations (Table 1). Interestingly, a small (1.2-fold) increase in GnRH-induced intracellular Ca^2+^ appears to occur in the presence of 10–100 pM antagonist, although this is not statistically significant, compared to treatment by GnRH alone.

SH-SY5Y and LβT2 cell lines were selected to serve as control models in vitro as they have been shown to endogenously express human [36] or mouse [35] GnRHRs, respectively. However, no GnRH-induced intracellular Ca^2+^ increase was mediated by the endogenous receptors in either of these cell lines (not shown). Overexpression of the GnRHR-encoding cDNA in the SH-SY5Y cells (SH-SY5Y/GnRHR cells) was required to achieve detectable signals. In this experimental setting, complete efficacy of GnRH antagonists is demonstrated at the 1 µM concentration (Supplementary Table S1) and no drug-specific differences were observed (Kruskal-Wallis test; *p* ≥ 0.05; Supplementary Figure S2). Unfortunately, transfection of LβT2 cells with GnRHR- and Ca^2+^ biosensor-encoding cDNA were not successful.

Both HEK293/GnRHR and SH-SY5Y/GnRHR control experiments, in which the effect of increasing antagonist concentrations was examined in the absence of GnRH stimulation, showed no response (Supplementary Figure S3).

### 2.2. Assessment of cAMP Accumulation

In order to find the optimal GnRH dose necessary to evaluate the action of antagonists in inhibiting the intracellular cAMP increase, dose-response experiments were performed. HEK293/GnRHR and SH-SY5Y cells transiently expressing the CAMYEL cAMP-biosensor were treated with increasing concentrations of GnRH (pM–µM range), in the presence of a phosphodiesterase inhibitor, and the 30 min cAMP accumulation was evaluated by BRET. The appropriate time-point for measurements of this second messenger was established after assessing the kinetics of the GnRH-induced cAMP accumulation, which is maintained at the plateau level between 10 and 50 min (Supplementary Figure S4). Similarly to what have been seen with Ca^2+^ accumulation, in untransfected SH-SY5Y cells, no GnRH-induced cAMP increases were detected (not shown), although these cells are reported to endogenously express the human GnRHR [36], as a likely effect due to cell-specific receptor expression levels and/or intracellular enzymatic milieu. Since no GnRH-induced Ca^2+^ accumulation was observed in LβT2 cells and endogenous GnRHRs are shown to be uncoupled from the Gα_s_ protein/cAMP/PKA-pathway in cells of nervous system origin [6], LβT2 cells were not used for these studies.

**Figure 3:**
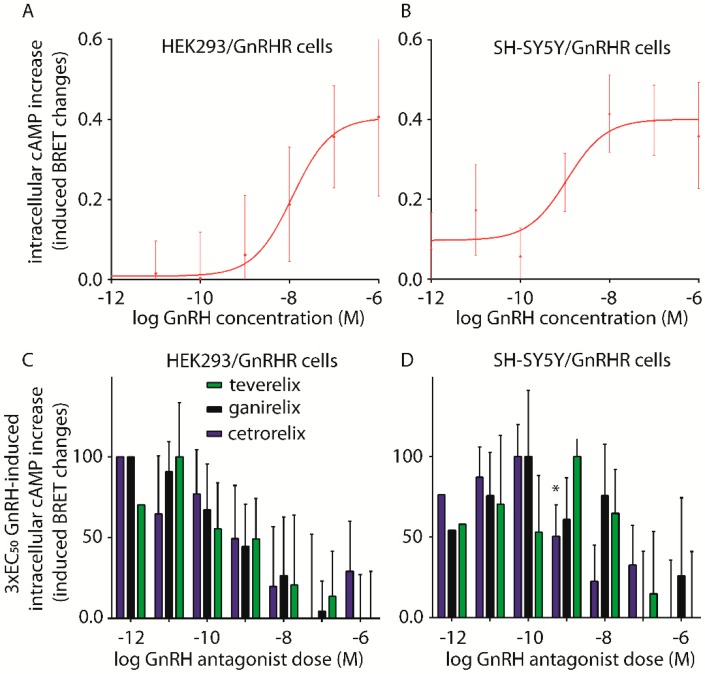
Evaluation of cAMP activation induced by GnRH, in the presence or in the absence of GnRH antagonists. (**A**) HEK293/GnRHR or (**B**) SH-SY5Y/GnRHR cells were maintained for 30 min with pM-µM GnRH concentrations, in the presence of 200 µM IBMX, as a phosphodiesterase enzymes inhibitor. AUC data are represented in the X-Y graph as means ± SEM and interpolated using non-linear regression. Dose-response curves obtained in the two cell models resulted in similar EC_50_s (HEK293/GnRHR = 11.58 ± 1.95 nM; *n* = 3; SH-SY5Y/GnRHR = 1.11 ± 4.05 nM; *n* = 5; Mann–Whitney U test; *p* ≥ 0.05; means ± SEM). (**C**,**D**) 3 × EC_50_ GnRH-induced cAMP inhibition by Cetrorelix, Ganirelix and Teverelix (pM–µM range), in (**C**) HEK293/GnRHR and (**D**) SH-SY5Y/GnRHR cells. cAMP data were interpolated by non-linear regression (not shown in panels C and D) and GnRH antagonist IC_50_s obtained in the two cell models were compared and found to be similar in HEK293/GnRHR cells (Cetrorelix = 0.84 ± 3.85 nM; Ganirelix = 0.61 ± 2.57 nM; Teverelix = 0.49 ± 3.21 nM; Mann–Whitney U test test; *p* ≥ 0.05; means ± SEM; *n* = 4), but not in SH-SY5Y/GnRHR cells (Cetrorelix = 1.56 ± 2.49 * nM; Ganirelix = 16.60 ± 3.76 nM; Teverelix = 62.80 ± 3.77 nM; * = significantly different versus Teverelix; Mann–Whitney U test; *p* < 0.05; means ± SEM; *n* = 5).

Treatment of HEK293/GnRHR and SH-SY5Y/GnRHR cells with GnRH-induced dose-dependent intracellular cAMP accumulation (Figure 3A,B). In both cell lines, similar GnRH potencies were observed (EC_50_s: HEK293/GnRHR = 11.58 ± 1.95 nM; *n* = 3; SH-SY5Y/GnRHR = 1.11 ± 4.05 nM; *n* = 5; Mann–Whitney U test; *p* ≥ 0.05; means ± SEM).

GnRH 3 × EC_50_ concentrations were then used as fixed concentrations for the evaluation of Cetrorelix, Ganirelix and Teverelix potencies in inhibiting the hormone-induced cAMP increase. Cells were treated with the hormone, in the presence of increasing antagonist concentrations, and intracellular cAMP accumulation was assessed. cAMP concentrations were determined as “induced BRET changes” and plotted against antagonist concentration in an X-Y graph before data interpolation by non-linear regression.

In HEK293/GnRHR cells (Figure 3C), similar dose-inhibition curves were obtained for each of the antagonists. These data demonstrate overall equal potency of antagonists in reducing the GnRH-induced cAMP accumulation (IC_50_s: Cetrorelix = 0.84 ± 3.85 nM; Ganirelix = 0.61 ± 2.57 nM; Teverelix = 0.49 ± 3.21 nM; Mann–Whitney U test test; *p* ≥ 0.05; means ± SEM; *n* = 4) and do not reflect the antagonist-specific modulation that was observed for calcium signaling in this cell line (Figure 2). Interestingly, in SH-SY5Y/GnRHR cells, Cetrorelix displayed higher potency than Teverelix or Ganirelix in inhibiting cAMP accumulation (Figure 3D), (IC_50_: Cetrorelix = 1.56 ± 2.49* nM; Ganirelix = 16.60 ± 3.76 nM; Teverelix = 62.80 ± 3.77 nM; * = significantly different versus other antagonists; Mann–Whitney U test; *p* < 0.05; means ± SEM; *n* = 5). Control experiments, in which cells were treated with GnRH antagonists in the absence of the hormone, demonstrated no cAMP activation (Supplementary Figure S5).

### 2.3. Evaluation of pERK1/2 and pCREB Activation

**Figure 4:**
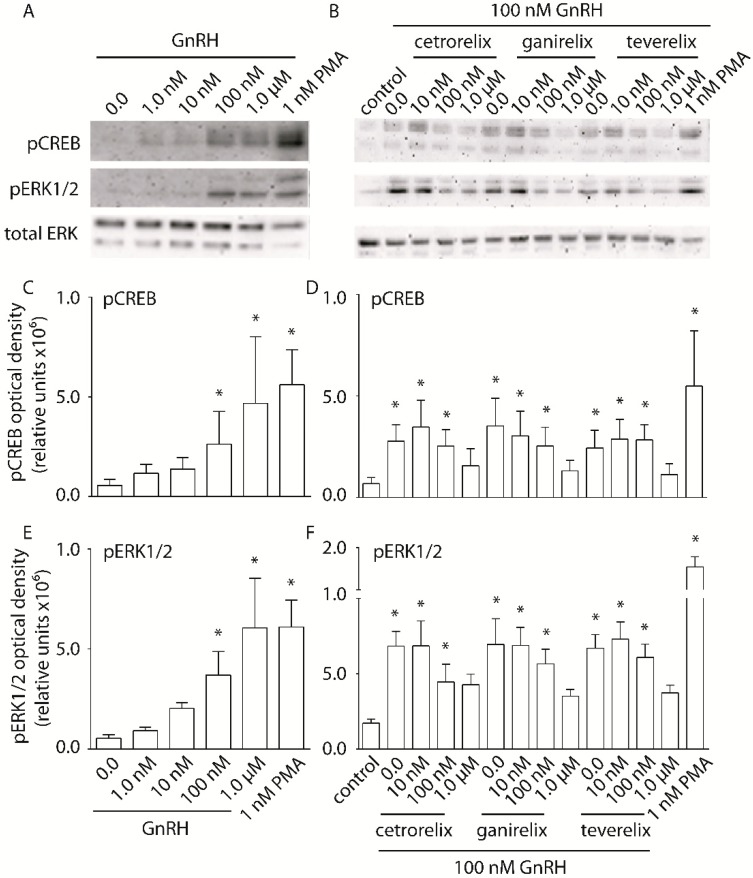
GnRH-induced pERK1/2 and pCREB activation, and inhibition exerted by GnRH antagonists, in HEK293/GnRHR cells. (**A**) Cells were treated 15 min with increasing GnRH concentrations (pM–µM range) and the phosphorylation of ERK1/2 and CREB was evaluated by Western blotting. Untreated samples are used as negative controls, while samples treated by 1 nM PMA served as positive control. Total ERK was the loading control. Images are representative of three independent experiments. (**B**) Cetrorelix, Ganirelix and Teverelix dose-inhibition of 100 nM GnRH-induced pERK1/2 and pCREB activation. Images are representative of three independent experiments. (**C**–**F**) Semi-quantitative evaluations of pCREB and pERK1/2 Western blotting signals displayed in panels A and B, by an image analysis software. Bars indicate means ± SEM (* = significantly different versus control; Kruskal-Wallis test; *p* < 0.05; *n* = 3).

The phosphorylation of ERK1/2 and CREB, as events occurring downstream of intracellular Ca^2+^ and/or cAMP accumulation, were evaluated in cells treated by nM–µM GnRH concentrations (Figure 4). HEK293/GnRHR cells were treated with GnRH, in the presence or in the absence of antagonists before evaluation of ERK1/2 and CREB phosphorylation by semi-quantitative Western blotting

HEK293/GnRHR cell treatment by 100 nM and 1 µM GnRH resulted in significant activation of both pERK1/2 and pCREB (Figure 4A), compared to basal levels (Kruskal-Wallis test; *p* < 0.05; *n* = 3). This reflects the hormone doses required for maximal intracellular Ca^2+^ and cAMP accumulation (Figure 2 and Figure 3).

Since 100 nM GnRH was the minimum concentration required to see robust activation of these phospho-proteins, it was used as a fixed dose for testing pERK1/2 and pCREB inhibition by the antagonists (Figure 4B). Inhibition of pCREB activation occurred upon treatment of cells with antagonist concentrations as low as 10 nM, with complete inhibition observed with concentrations of 1 µM (Kruskal-Wallis test; *p* < 0.05; *n* = 3). Inhibition of GnRH-induced pERK1/2 activation required higher doses of antagonist, but at 1 µM, significant inhibition was observed. The inhibition profiles of each antagonist were similar, revealing that Cetrorelix, Ganirelix and Teverelix similarly impact the phospho-protein activation. One representative experiment performed with gonadotrope LβT2 cells revealed similar findings (Supplementary Figure S6), while sub-optimal Western blotting signals were obtained using SH-SY5Y/GnRHR cells, which impeded data interpretation (not shown).

### 2.4. Analysis of β-Catenin Activation and Localization

In HEK293/GnRHR cells, activation and intracellular localization of β-catenin in response to GnRH treatment was assessed by fluorescence microscopy. Cells were treated for 1 h with a high concentration (1 µM) of GnRH, in the presence or absence of 1 µM GnRH antagonist, prior to fixation and staining with an anti-active-β-catenin antibody and DAPI nuclear stain. Cells treated for 16 h with 100 nM CHIR99021 served as a positive control for β-catenin activation.

**Figure 5:**
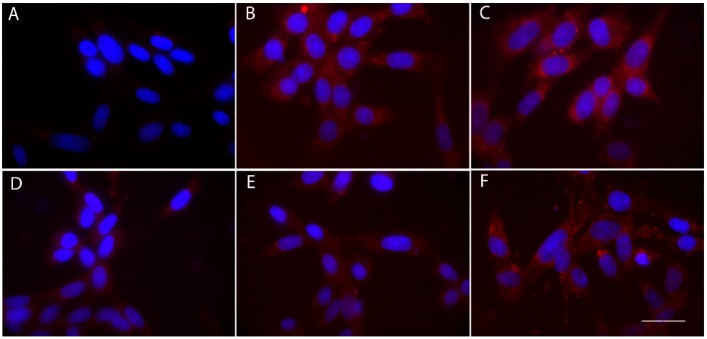
GnRH-induced β-catenin activation and inhibition by antagonists, in HEK293/GnRHR cells. Cells were treated for 1 h by 1 µM dose of GnRH, in the presence or in the absence of 1 µM GnRH antagonist, and immunostaining was performed in fixed samples using a primary antibody against the active-β-catenin and a Cy3-conjugated secondary antibody (red). DAPI was used for nuclei staining (blue). (**A**) Control samples maintained in the absence of hormone and GnRH antagonist (negative control). (**B**) Cells treated with GnRH alone, (**C**) Cells treated for 16 h with 100 nM of the glycogen synthase kinase 3 (GSK3) inhibitor CHIR99021 cells (positive controls for β-catenin activation). (**D**–**F**) Cells treated with (**D**) both GnRH and Cetrorelix, (**E**) Ganirelixor and (**F**) Teverelix. Images are representative of three independent experiments. Bar = 50 µm.

While no active β-catenin staining (red in Figure 5) was observed in untreated cells, 1 h treatment with GnRH resulted in robust activation and staining of the molecule, detectable even in the nuclei (Figure 5). Similar to that demonstrated using the positive control CHIR99021, GnRH-activated β-catenin was localized both in the cytoplasm and in the nucleus of the cell. This staining was reduced in the presence of 1 µM Cetrorelix or Ganirelix, while the equimolar concentration of Teverelix did not result in any decrease of signal.

### 2.5. Analysis of GnRH-Induced Lhb Gene Expression

One of the physiological outcomes of GnRHR-mediated signaling in pituitary gonadotropes is the increased expression and secretion of the gonadotropins, luteinizing hormone (LH) and follicle-stimulating hormone (FSH). To assess the potential effects of the different antagonists’ gonadotropin expression, *Lhb* gene expression was evaluated by qRT-PCR in LβT2 cells. The same analysis was not possible in HEK293 and SH-SY5Y due to the lack of LH receptor-encoding gene expression. Cells were first treated by increasing doses of GnRH (nM–µM range) to find the optimal GnRH concentration to be used for subsequent experiments comparing Cetrorelix, Ganirelix and Teverelix efficacy in inhibiting *Lhb* gene transcription. 

**Figure 6:**
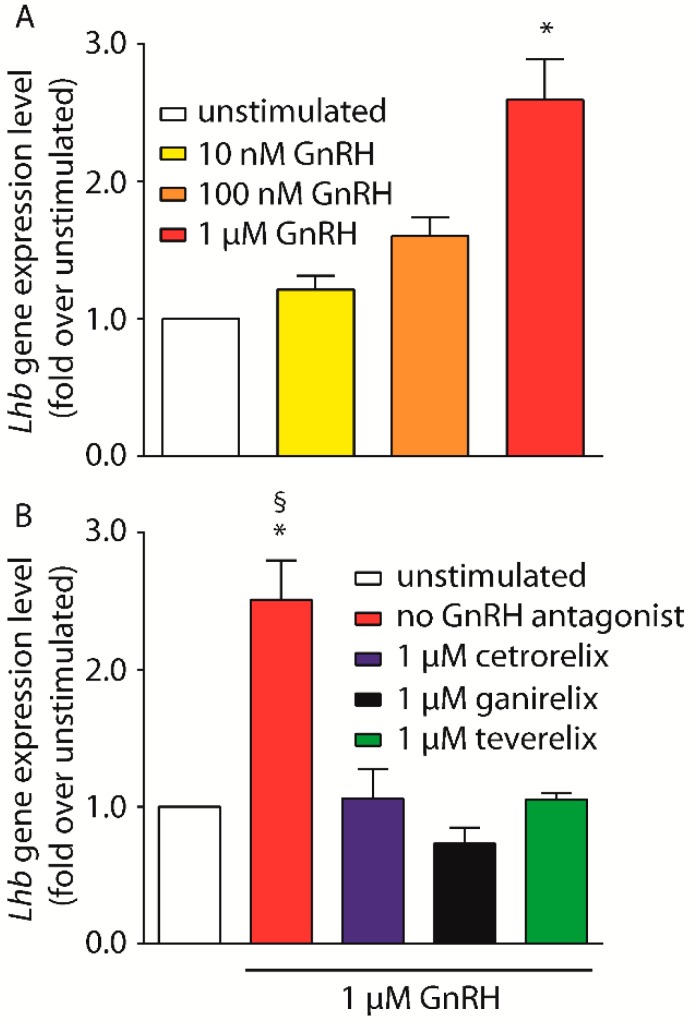
*Lhβ* gene expression induced by GnRH, in LβT2 cells. (**A**) Samples were treated for 24 h with increasing doses of GnRH (nM–µM range) and *Lhb* gene expression levels detected by real time PCR. Data were normalized to *Gapdh* gene expression levels and are presented as fold-increase over basal (unstimulated cells). (**B**) Results from real time PCR analysis of LβT2 cells treated with 1 µM GnRH in the presence of 1 µM of Cetrorelix Ganirelix or Teverelix antagonist. * = significantly different versus unstimulated; ^§^ = significantly different versus antagonists; Kruskal-Wallis test; *p* < 0.05; means ± SEM; *n* = 4.

LβT2 cell treatment by 1 µM GnRH resulted in *Lhb* gene expression increase (2.6-fold over basal; Kruskal-Wallis test; *p* < 0.05; *n* = 4), while lower hormone concentrations did not result in significantly different expression levels compared to basal (Figure 6A).

1 µM was therefore used as a fixed GnRH concentration to test the efficacy of equimolar concentrations of antagonists in inhibiting *Lhb* gene expression. 1 µM of Cetrorelix, Ganirelix or Teverelix inhibited GnRH-induced activation of target gene expression (Figure 6B) (Kruskal-Wallis test; *p* < 0.05; *n* = 4), achieving *Lhb* expression levels similar to basal (Kruskal-Wallis test; *p* ≥ 0.05; *n* = 4).

## 3. Discussion

Herein, it has been demonstrated that three GnRH antagonists developed for clinical purposes, such as prevention of premature ovulation and prostate cancer therapy [37], have different potencies in inhibiting hormone-dependent intracellular signaling in vitro. Peptide chains of Cetrorelix, Ganirelix and Teverelix differ by two amino acids [5,26], likely specifically modulating their receptor binding and GnRH-induced signaling. Indeed, 10 nM Cetrorelix fully inhibited intracellular Ca^2+^ increase upon HEK293/GnRHR cell treatment by the hormone, while the same concentration of the other two antagonists resulted in only partial antagonism. These data are supported by the higher potency of Cetrorelix in inhibiting cAMP production in SH-SY5Y/GnRHR cells, although all three antagonists elicit equivalent inhibition on GnRH-induced cAMP responses in HEK293/GnRHR cells in vitro. Furthermore, inhibition of GnRH-induced activation of β catenin by GnRH in HEK293/GnRH cells was inhibited by Cetrorelix and Ganirelix but not Teverelix. Taken together, these data suggest that GnRH antagonists are capable of cell-specific, biased signaling. Other downstream intracellular signals, such as pERK1/2 and pCREB activation, were not affected by antagonist-specific inhibition and hormone-induced expression of the *Lhb* target gene was decreased similarly by all three, in the mouse LβT2 cell line.

Amino acidic differences between these GnRH antagonists fall within a peptide region which is crucial in regulating the interaction with the GnRHR [5,38]. In particular, Cetrorelix carries the amino acid arginine at position 8 of the protein chain (Figure 1), where Ganirelix and Teverelix have the D-isomer of arginine (D-arginine) or lysine, respectively. In native human GnRH there is an arginine in this position which is conserved in mammals (although less conserved in other vertebrates [39]). This arginine has been proposed to be a contact point with an asparagine residue located within the third extracellular loop of the receptor, and to play a role in stabilizing the receptors active conformation and thus its activation [34]. Cetrorelix and Teverelix both have a D-isomer of citrulline at position 6 (Figure 1), while Ganirelix carries a D-arginine in this position [5]. In vertebrates, native GnRH peptides display a highly conserved glycine at position 6. This residue plays a crucial role in preserving binding affinity to the human receptor and in stimulating activation and down-stream signaling, such as inositol phosphate production [39]. 

GnRH-induced intracellular Ca^2+^ increase was evaluated by BRET in transfected cell lines overexpressing a specific biosensor and GnRHR. Previous studies demonstrated that, in pituitary gonadotrope cells, calcium signalling is potently up-regulated by GnRH via activation of ion channels [40,41] and it is a fundamental mediator of the LH and FSH release [42,43]. In fact, in LβT2 cells, *Lhb* mRNA expression, induced by GnRH agonists, can be inhibited by a Ca^2+^ chelator [44]. However, in the present study, no consistent GnRH-induced intracellular Ca^2+^ increase was observed in LβT2 cells, presumably due to relatively low transfection and transcription efficiency of the Ca^2+^ biosensor-encoding plasmid. Experiments performed using transfected HEK293 and SH-SY5Y cell lines overexpressing both the biosensor and the hormone receptor, revealed that intracellular Ca^2+^ increase may be dampened, or even prevented by nM–µM concentrations of GnRH antagonist. Interestingly, we demonstrated that Cetrorelix has higher potency than Ganirelix and Teverelix in inhibiting calcium signaling. 10 nM Cetrorelix fully antagonizes 3 × EC_50_ GnRH, while other compounds fully antagonize the hormone only when they are administered at higher concentrations. These different inhibitory potencies may be assumed to be a result of compound-specific intracellular signaling signatures underlying the LiSS concept [34] and provide a basis to explain apparent differential clinical effects, such as lower pregnancy rate with Ganirelix versus Cetrorelix when using these antagonists for ART [30]. This data also supports other in vitro studies, which have revealed that GnRH antagonists may counteract hormone-induced apoptosis in GnRHR-expressing cells [45] and this effect may vary according to the type of antagonist used [46].

It is noteworthy that at pM concentrations, the antagonists appeared to potentiate rather than inhibit GnRH-induced intracellular Ca^2+^ accumulation, although these effects were not statistically significant. Agonistic action exerted by low GnRH antagonist concentrations has previously been described using human prostate cancer cells [46]. However, the molecular mechanism underlying this behavior is not known.

The primary transduction mechanism utilized by the GnRHR is via coupling to Gα_q/11_ G proteins. Coupling to the cAMP pathway has also been observed but this has been attributed to the overexpression of exogenous receptor in transfected cells [47], since it typically does not occur in human gonadotrope cells [5]. Indeed, cAMP production may be achieved with non-human GnRHR due to a specific motif in the C-terminal tail [48], which is lacking in the human receptor [49]. Considering together intracellular Ca^2+^ and cAMP data obtained from HEK293/GnRHR cells, it is conceivable that antagonists mediate biased GnRH-induced signaling, strengthening the concept that they may be linked to LiSS [34]. Indeed, cell treatment by Cetrorelix, Ganirelix and Teverelix resulted in similar cAMP inhibition, in spite of higher potency of Cetrorelix in depleting the hormone-induced intracellular Ca^2+^ increase. Interestingly, although effects on intracellular Ca^2+^ accumulation were similar in the HEK293/GnRH and SH-SY5Y cells, antagonist effects on GnRH-induced cAMP accumulation were different between the two cell lines. In the SH-SY5Y cells, Cetrorelix displayed higher efficacy than other compounds in inhibiting GnRH-induced cAMP increase, reflecting the effects on intracellular Ca^2+^ accumulation. These cell line-specific differences might be due to cell-specific receptor interacting proteins [50], resulting in the so-called tissue bias [51].

Analysis of GnRH-induced pCREB and pERK1/2 activation suggested that the activity of these cAMP-dependent kinase enzymes [52] is similarly modulated by all three antagonists, which were equipotent in decreasing the phosphorylation of ERK and CREB, corroborating with the findings from the cAMP accumulation studies, at least in the HEK293/GnRHR cells. Unfortunately, comparisons between cAMP and phospho-protein data obtained from SHSY5Y/GnRHR were not possible due to the inconsistency of Western blotting signals (not shown), while results from gonadotrope LβT2 cells confirmed that in this cell line, the GnRH antagonists are also equipotent in inhibiting pERK1/2 and pCREB activation. Since phospho-proteins control the GnRH pulse frequency [52] and transcription of gonadotropin β subunits gene [53,54], we might expect that Cetrorelix, Ganirelix and Teverelix similarly impact *Lhb* gene mRNA levels in LβT2 cells. The transcription of GnRH target genes is modulated by several factors, such as β-catenin [24], which activation, in turn, is itself dependent on a number of molecules including intracellular Ca^2+^ and CREB. Although β-catenin activation acts as an enhancer of the *Lhb* gene transcription [55], and immunostaining has revealed its weak inhibition by Teverelix, all three antagonists had similar effects on GnRH-induced *Lhb* expression. These data highlight the potential of GnRH antagonists in exerting LiSS behavior at the early signaling level, in spite of the similar inhibition of gonadotropin production. This observation of differential effects at different endpoints may be due to the balance of GnRH-mediated signals at the intracellular level, as previously demonstrated by treating gonadal cells by gonadotropic hormones [56,57], resulting in smoothing of the ligand-specific, early biased signaling across the intracellular cascade progression. While future studies could examine these effects further using human gonadotrope cell lines and also further explore the links to clinical efficacy, the data obtained herein are overall indicative of the structure-specific action of the GnRH antagonists at the receptor. On the other hand, limitations due to the in vitro environment and transcription efficiency of the receptor-encoding cDNA used for cell transfection should be taken into account, since it might result in cell-specific responses. Therefore, it may be supposed that the cell model may be a source of variations between activities of antagonists due to intrinsic factors, such as GnRHR expression levels, exposing the cell context to receptor saturation at high hormone concentrations or other potential modulatory effects to be further investigated.

## 4. Materials and Methods 

### 4.1. Materials

Cell treatments were performed using GnRH purchased from SANOFI (Relefact LH-RH 0.1 mg), in the presence or in the absence of Cetrorelix acetate (C5249; Sigma-Aldrich, St. Louis, MO, USA), Ganirelix acetate (SML024; Sigma-Aldrich) or Teverelix [31]. Hemagglutinin-tagged GnRHR (GnRHR-HA)-expressing vector was previously described [58] and was used for inducing the overexpression of the receptor in cell lines.

### 4.2. Cell Culture

Human embryonic kidney (HEK293) and mouse pituitary gonadotrope LβT2 cells [35] were grown in DMEM medium, modified with 4.5 g/L glucose and supplemented with 10% fetal bovine serum (FBS), 2 mM glutamine, 100 U/mL penicillin and 0.1 mg/mL streptomycin (Invitrogen, Carlsbad, CA, USA). Human neuroblastoma-derived SH-SY5Y cells were kindly provided by Professor Fabio Tascedda (University of Modena and Reggio Emilia, Modena, Italy) and maintained in DMEM/F12 medium, supplemented with 10% FBS, 2 mM glutamine, 100 U/mL penicillin, 0.1 mg/mL streptomycin and 1% MEM non-essential amino acids solution (Invitrogen, Carlsbad, CA, USA). All cell lines were maintained at 37 °C and 5.0% CO_2_. 

While the mouse and human GnRHR are known to be naturally expressed in both the LβT2 [35] and SH-SY5Y cell lines [36] respectively, they are not in HEK293 cells.

### 4.3. Transfection Methods

Different transfection protocols were followed, depending on the endpoint to be measured. For BRET experiments, 3 × 10^4^ cells were transiently transfected using vectors expressing the cAMP-CAMYEL or the aequorin-Ca^2+^ BRET biosensor-encoding cDNA [59], as appropriate, together with the GnRHR-HA-encoding vector. Transfections were performed in a 96-well plate using the Metafectene PRO reagent (Biontex, München, Germany), as previously described [60]. 100 ng of the receptor-encoding vector + 100 ng of the BRET biosensor-encoding vector were used for co-transfecting HEK293 cells, while SH-SY5Y cells were co-transfected using 200 ng of the receptor-encoding vector + 100 ng of the BRET biosensor-encoding vector. 200 ng of the aequorin-Ca^2+^ BRET biosensor-encoding cDNA were used for transfecting LβT2 cells. Each sample was prepared in duplicate, cultured for 48 h and serum-starved over-night before experiments. 

In order to evaluate the activation of phospho-proteins and β-catenin by Western blotting and immunofluorescence, 1 × 10^5^ HEK293 cells were transiently transfected in a 24-well plate. To this purpose, 500 ng of GnRHR-HA-encoding vector diluted in 2 µL/well Lipofectamine® 2000 Transfection Reagent (Invitrogen) were used, according to the manufacturer’s instructions. Transfected HEK293 cells (HEK293/GnRHR) were cultured for 48 h and maintained over-night in serum-free medium before experiments.

### 4.4. BRET Measurement of Intracellular Ca^2+^ Increase and cAMP Production

GnRH-induced intracellular cAMP and Ca^2+^ increase was assessed by the BRET technique in biosensor- and GnRHR-expressing cells, as previously described [57,59,61,62], in the presence or in the absence of GnRH antagonist. To evaluate Ca^2+^ increase, cells were incubated in Hank’s Balanced Salt Solution (HBSS) with Ca^2+^ and Mg^2+^, 1 mM Hepes and 5 µM Coelenterazine H (NanoLight Technologies, a division of Prolume, Ltd., Pinetop, AZ, USA), for 45 minutes, together with Cetrorelix, Ganirelix, Teverelix or vehicle, as appropriate. Light emissions at 480 and 540 nm wavelengths were detected every 1.68 s, over 150 s, while a fixed concentration of GnRH (equivalent to the 50% effective concentration (EC_50_)) was injected at the 21.94 s time-point. Dose-response experiments were performed by treating LβT2, HEK293/GnRHR and SH-SY5Y/GnRHR cells with increasing concentrations of GnRH (pM-µM range) in the absence of antagonist. AUC extrapolated from Ca^2+^ kinetics was calculated, for each cell line, and plotted against the GnRH concentration in a X-Y graph. The EC_50_ was extrapolated from the curve obtained from each cell line. Cells treated by 5 µM of the sarco/endoplasmic reticulum Ca^2+^ ATPase enzyme inhibitor, thapsigargin (Tocris Bioscience), served as positive control [63]. Treatment by the vehicle (HBSS) was the negative control and used as a normalizer.

cAMP accumulation was measured in cells transiently expressing both the BRET-based cAMP biosensor CAMYEL [64] and GnRHR. Dose-response experiments were prepared by 20 min cell incubation in 40 µL/well of Dulbecco’s phosphate buffered saline (PBS), 1 mM HEPES and 200 µM of the phosphodiesterases inhibitor 3-isobutyl-1-methylxanthine (IBMX) (#I5879, Sigma-Aldrich) before 30 min treatment by increasing doses of antagonist together with a fixed (3 × EC_50_) concentration of GnRH. BRET signals were detected at 475 and 535 nm wavelengths upon the addition of 5 µM Coelenterazine H (NanoLight Technologies). Treatment by 200 µM of the adenylyl cyclase activator forskolin (Sigma-Aldrich) served as a positive control.

BRET experiments were performed using a CLARIOstar plate reader equipped by a monocromator (BMG Labtech, Ortenberg, Germany), as previously described [56,59,60,61,62,63].

### 4.5. Western Blotting Analysis

GnRH-induced ERK1/2 and CREB phosphorylation, in the presence or in the absence of antagonists, was analyzed by Western blotting, as previously described [65,66]. LβT2 and HEK293/GnRHR cells were plated in 24-well plates (1 × 10^5^ cells/well). After 48 h, cells were incubated in serum-free media for 24 h and treated for 15 min with GnRH in the presence or absence of antagonists. 20 nM of the activator of protein kinase C (PKC), phorbol 12-myristate 13-acetate (PMA), was used as a positive control [65]. After treatment, cells were lysed in ice-cold RIPA buffer enriched by one tablet/10 mL of the phosphatase inhibitor PhosStop (Roche, Basel, Switzerland) and protease inhibitor cocktail. pERK1/2 and pCREB were detected using specific rabbit antibodies (#9101and #9198, respectively; Cell Signaling Technology Inc., Danvers, MA, USA) after Western blotting, while total ERK1/2 (# 4695; Cell Signaling Technology Inc.) served as a normalizer. Membranes were incubated with a secondary anti-rabbit HRP-conjugated antibody (#NA9340V; GE HealthCare, Chicago, IL USA) before signal development through the ECL chemiluminescent compound (GE HealthCare) and acquisition by the QuantityOne analysis software (Bio-Rad Laboratories Inc., Hercules, CA, USA). Densitometric analyses were performed using the ImageJ software (National Institute of Health, Bethesda, MD, USA) [67].

### 4.6. Immunofluorescence Analysis

3 × 10^4^ HEK293/GnRHR and mock-transfected HEK293 cells (negative control) were plated on gelatine-treated slides. After 48 h, cells were serum starved for 16 h before 1 h treatment by 1 µM GnRH, in the presence or in the absence of equimolar concentrations of the antagonists. As a positive control, cells were treated for 16 h with 100 nM of the selective glycogen synthase kinase 3 (GSK3) inhibitor, CHIR99021 (Sigma-Aldrich), which stabilizes free cytosolic β-catenin [68]. After stimulations, cells were washed in PBS with Ca^2+^/Mg^2+^ and fixed for 20 min in paraformaldehyde 4%/PBS. Cell monolayers were washed by PBS and permeabilized using 0.1% Triton X-100. Samples were incubated in 4% bovine serum albumin (BSA)/PBS, for 1 h at room temperature (RT), before incubation with a mouse primary antibody targeting β-catenin (#05-665, 1:300 dilution in 4% BSA/PBS; Merck KGaA) overnight at 4 °C. Following washing, cells were then incubated with Cy3-conjugated anti-mouse IgG secondary antibody (#C2181, 1:200 dilution in 4% BSA/PBS; Sigma-Aldrich) for 1 h at RT. Cell monolayers were then stained for 10 min with 4′,6-diamidino-2-phenylindole (DAPI) (Sigma-Aldrich) and washed in PBS before slide treatment by polyvinyl alcohol mounting medium with 1,4-diazabicyclo[2.2.2]octane (DABCO^®^) (Sigma-Aldrich). Fluorescent signals were detected using the Eclipse Ni-E microscope (Nikon Instruments BV, Amsterdam, The Netherlands).

### 4.7. Lhb Gene Expression

LβT2 cells were seeded in 24-well plates (2 × 10^5^ cells/well) and cultured for 24 h in DMEM supplemented with 10% Charcoal Stripped FBS, 4.5 g/L glucose, 100 IU/mL penicillin, 0.1 mg/mL streptomycin, and 1 mM glutamine (all from Sigma-Aldrich) before treatments. Cells were maintained for 24 h with increasing GnRH concentrations (nM–µM range) in the presence or absence of antagonist. Samples were lysed for RNA extraction using TRIzol™ Reagent (Invitrogen) and equal amounts of total RNA were used for cDNA synthesis performed using the iScript reverse transcriptase (BioRad), as previously described [57]. qRT-PCR was carried out in triplicates on the CFX96™ Real-Time PCR Detection System (BioRad) using the following primers pairs: fwd: 5′-CTGGCCGCAGAGAATGAGTT-3′; rev:5′-TCGGACCATGCTAGGACAGT-3′ for mouse *Lhb*; 5′-GGCATTGCTCTCAATGACAA-3′; rev:5′-ATGTAGGCCATGAGGTCCAC-3′ for mouse *Gapdh*. Primer sequences were drawn using the *Mus musculus Lhb* (NCBI Reference Sequence: NM_008497.2) and *Gapdh* (NM_008084.3) mRNAs as templates. Gene expression of *Lhb* was normalized to mouse *Gapdh* gene expression using the 2^−ΔΔCt^ method [69].

### 4.8. Statistical Analysis 

Data were plotted on graphs as means ± standard error of means (SEM). The kinetics of intracellular Ca^2+^ increase was represented by the BRET biosensor emission after background subtraction (vehicle). AUC were calculated for each line and plotted against the GnRH concentration, obtaining dose-response curves after non-linear regression analysis. Data distributions were analyzed by D’Agostino and Pearson normality tests and inhibition of intracellular Ca^2+^ increase by different concentrations of antagonist were compared using two-way analysis of variance (ANOVA), followed by Bonferroni post-test or Kruskal-Wallis and proper post-test. cAMP accumulation was represented as acceptor:donor emission ratio (induced BRET changes) after basal subtraction (untreated cells). Dose-response curves were obtained by data interpolation using non-linear regressions and normalized as percentage of the maximal response. EC_50_s, as well as half-maximal inhibitory concentrations (IC_50_), were compared by t-test or Mann–Whitney U test, while results obtained by Western blotting and qRT-PCR data were compared by one-way ANOVA followed by Bonferroni post-test. Statistical differences were considered as significant for *p* < 0.05. These analyses were performed using the GraphPad Prism software (GraphPad Software Inc., San Diego, CA, USA). 

## 5. Conclusions

Cetrorelix, Ganirelix and Teverelix are characterized by different amino acid structures which result in compound-specific differential potencies in inhibiting GnRH-mediated early signaling. Although their action may vary depending on cell type and GnRHR expression levels, Cetrorelix displays the highest efficacy in depleting intracellular Ca^2+^ and cAMP increase, while Teverelix has the lowest potency in depleting hormone-induced β-catenin activation and all three antagonists show similar effects on phospho-protein activation. Nevertheless, biased signaling demonstrated between the antagonists at earlier endpoints suggests that structure-specific features of GnRH antagonists are linked to LiSS behavior. However, these differences observed in intracellular signaling cascades are not reflected in compound-specific inhibition of the *Lhb* gene transcription, a final physiological result of GnRH signaling in gonadotrope cells, as all three antagonists had equivalent effects. These data provide a molecular basis for evaluating whether GnRH antagonists, used for depleting the production of pituitary gonadotropins, are linked to potentially different clinical outcomes which may be relevant for the clinical use of these drugs and future development of similar analogues.

## Figures and Tables

**Table 1:** GnRH-antagonist dose-dependent AUCs of the kinetics of intracellular Ca^2+^ response.

No Antagonist	109,340 ± 13,866		
	Cetrorelix	Ganirelix	Teverelix
1 pM	78,113 ± 29,979	76,189 ± 18,564	85,301 ± 21,321
10 pM	124,981 ± 33,684	91,896 ± 21,766	98,939 ± 27,373
100 pM	126,243 ± 34,982	101,943 ± 27,388	58,864 ± 27,101
1 nM	71,127 ± 25,268	66,665 ± 16,035	81,702 ± 18,920
10 nM	21,482 ± 6718 *	**73,164 ± 16,237**	**74,321 ± 17,569**
75 nM	18,632 ± 4423 *	43,870 ± 4193 *	42,401 ± 8974 *
100 nM	8946 ± 3296 *	34,734 ± 13,731 *	29,931 ± 8731 *
1 µM	3264 ± 3024 *	3346 ± 2977 *	4107 ± 3804 *

Bold: Kruskal-Wallis test and Dunn’s post-test after correction for multiple comparisons (means ± SEM; *p* < 0.05; *n* = 6). * Significant versus no antagonist.

## Supplementary Materials

Supplementary materials can be found at https://www.mdpi.com/1422-0067/20/22/5548/s1.
