# Supplementary material for: GnRH Antagonists Produce Differential Modulation of the Signaling Pathways Mediated by GnRH Receptors
Source: Int J Mol Sci. 2019 Nov 7;20(22):5548. doi: 10.3390/ijms20225548 (PMC6888270; doi:10.3390/ijms20225548)
Supplement: Supplementary file 1 [file ijms-20-05548-s001.zip › Supplementary Figure captions.docx]

**Supplementary Figure captions**

**Supplementary figure 1.** **Dose-response analysis of GnRH-induced intracellular Ca^2+^ increase, in SH-SY5Y/GnRHR and LβT2 cells, in the presence or in the absence of GnRH and antagonists**. A) SH-SY5Y/GnRHR or B) LβT2 cells were treated with increasing GnRH concentrations (pM-µM range) and Ca2+ BRET biosensor signal was measured over 150 s, with hormone injection occurring at the 20 s time-point. AUCs were calculated from kinetic data and represented as means±SEM. The dose-response curve was obtained by interpolating data using non-linear regression (EC50=23.26±3.37 nM; means±SEM; n=4).

**Supplementary figure 2.** **Analysis of the kinetics of GnRH-induced intracellular Ca^2+^ increase, in SH-SY5Y/GnRHR cells, in the presence or in the absence of GnRH and antagonists**. Ca^2+^ BRET biosensor signal was measured over 150 s. Kinetics of GnRH-induced intracellular Ca^2+^ increase, in the presence or in the absence of pM-µM concentrations of Cetrorelix (**A**), Ganirelix (**B**) and Teverelix (**C**), is indicated as light emissions and represented in the X-Y graph (means±SEM; n=4; consecutive points were connected by lines). The time of 3xEC_50_ GnRH injection is indicated by arrows.

**Supplementary figure 3.** **Analysis of the kinetics of GnRH antagonist-induced intracellular Ca^2+^ increase, in HEK293/GnRHR cells, in the absence of GnRH**. 150-s kinetics of Ca^2+^ intracellular increase, potentially consisting in background signal due to injection of pM-µM concentrations of **A**) Cetrorelix, **B**) Ganirelix and **C**) Teverelix, in the HEK293/GnRHR cell line. Experiments were performed in the absence of GnRH, while arrows show the antagonist injection time. Ca^2+^ is indicated as light emissions and represented in the X-Y graph (means±SEM; n=6; consecutive points were connected by lines).

**Supplementary figure 4.** **Analysis of the kinetics of GnRH-induced intracellular cAMP increase, in HEK293/GnRHR cells.** Timing of GnRH dose-response curves (figure 3) was optimized after evaluating cAMP BRET biosensor signal over 3000 s, in the presence or in the absence of 1 µM GnRH. Kinetics of GnRH-induced intracellular cAMP increase is indicated as induced BRET changes and represented in the X-Y graph (means±SEM; n=4). Data were normalized by basal subtraction and interpolated by *non*-linear regression. Experiments were performed in the presence of IBMX, while samples treated with 200 µM forskolin served as positive controls.

**Supplementary figure 5. Evaluation of** **background** **intracellular cAMP increase by GnRH antagonist dose-response experiments, in HEK293/GnRHR and SH-SY5Y/GnRHR cells. A, C, E**) HEK293/GnRHR cells or **B, D, F**) SH-SY5Y/GnRHR cells were treated with increasing concentrations of **A, B**) Cetrorelix, **C, D**) Ganirelix and **E, F**) Teverelix, in the absence of GnRH, and the 30-min intracellular cAMP accumulation was measured. Since data were represented as *non*-normalized, 540/460 nm light signal ratio (means±SEM; n=5), decreasing signal occurs together with cAMP increasing. Experiments were performed in the presence of IBMX, while samples treated with 200 µM forskolin served as positive controls.

**Supplementary figure 6. GnRH-induced pERK1/2 and pCREB activation, and inhibition exerted by GnRH antagonists, in the LβT2 cell line**. **A)** Cells were treated 15 min with increasing GnRH concentrations (pM-µM range), and pERK1/2 and pCREB activation was evaluated by Western blotting. Untreated samples are used as negative controls, while samples treated by 1 nM PMA served as positive control. Total ERK was the loading control. Images are representative of three independent experiments. **B)** Cetrorelix, Ganirelix and Teverelix dose-inhibition of 100 nM GnRH-induced pERK1/2 and pCREB activation. Images are representative of three independent experiments.
