# Supplementary figures and images for: GnRH Antagonists Produce Differential Modulation of the Signaling Pathways Mediated by GnRH Receptors
Source: Int J Mol Sci. 2019 Nov 7;20(22):5548. doi: 10.3390/ijms20225548 (PMC6888270; doi:10.3390/ijms20225548)

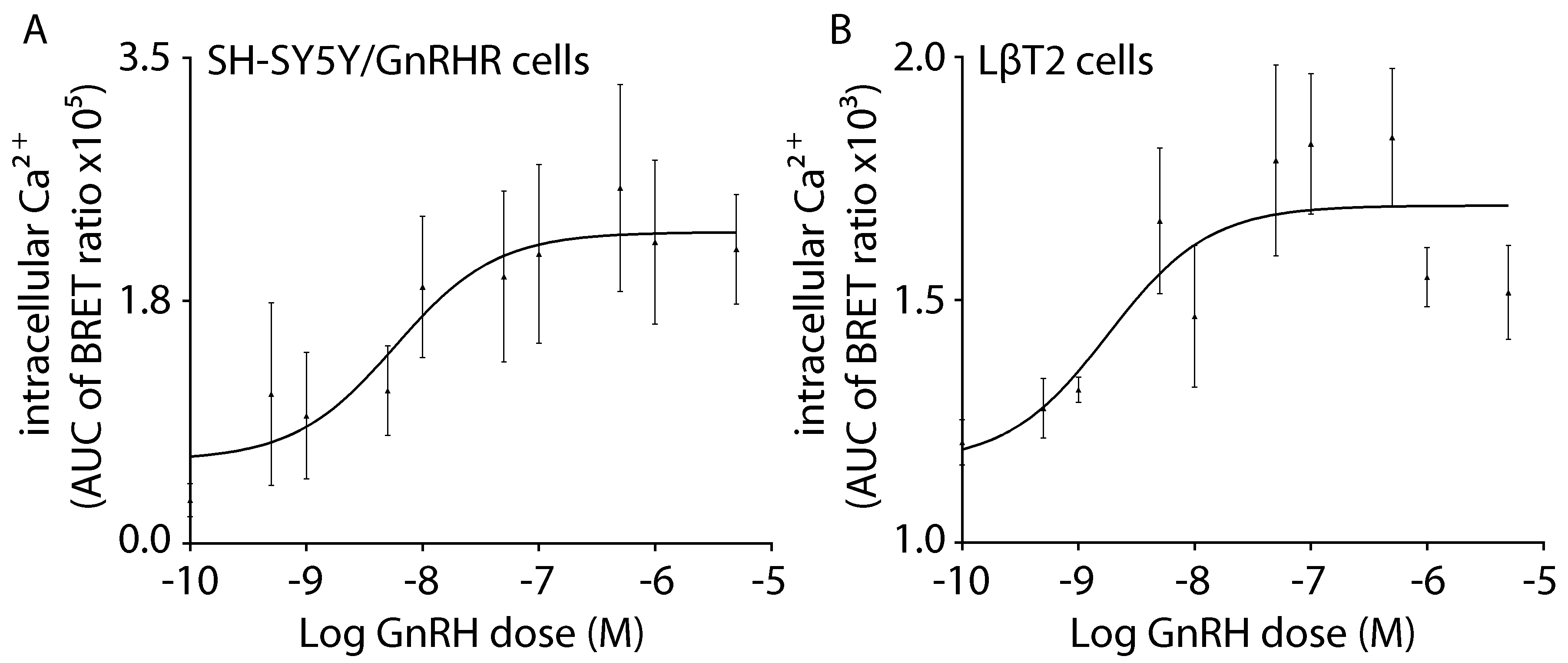

Supplement: Supplementary file 1 [file ijms-20-05548-s001.zip › Figure S1.tif]

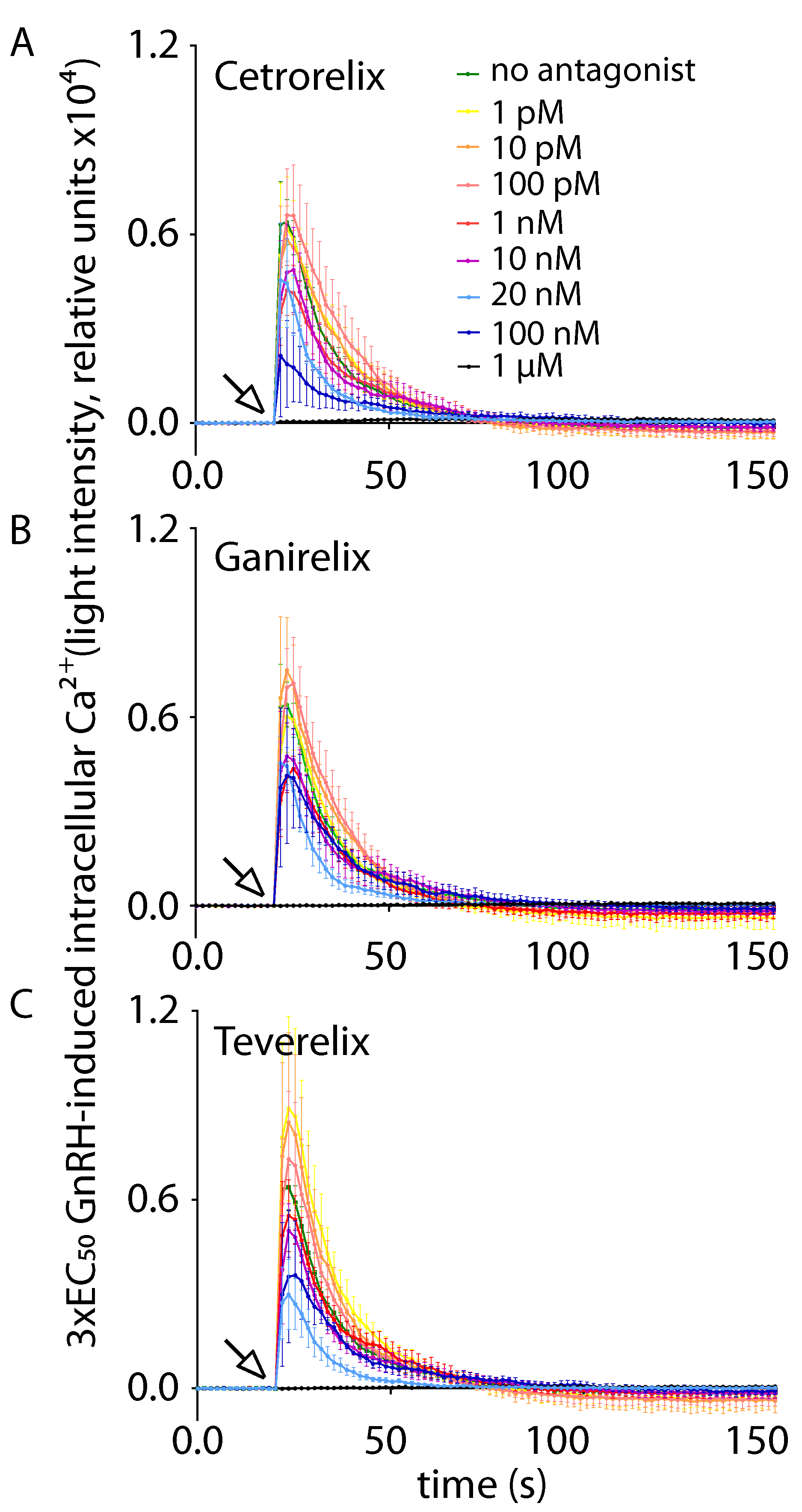

Supplement: Supplementary file 1 [file ijms-20-05548-s001.zip › Figure S2.tif]

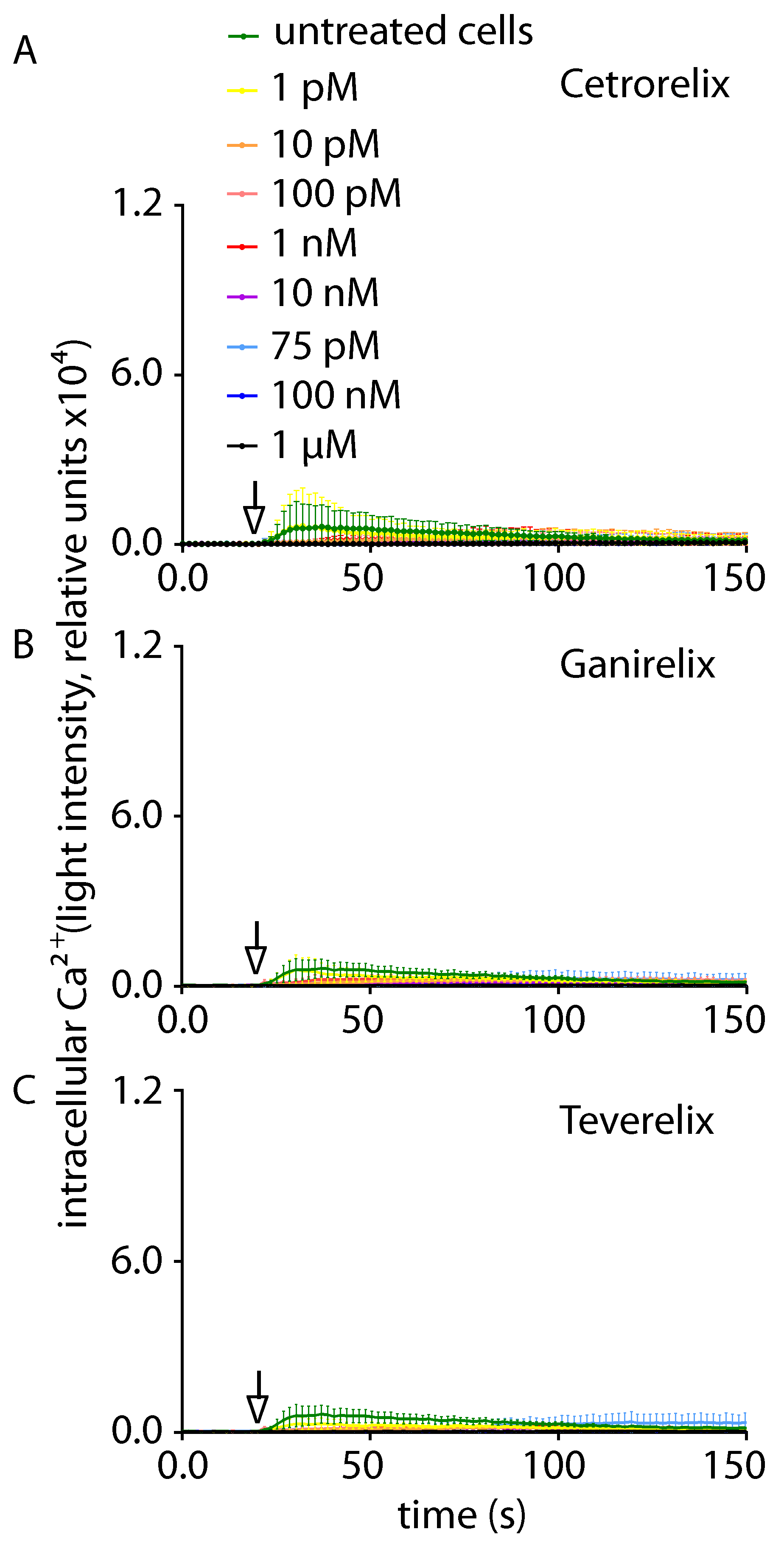

Supplement: Supplementary file 1 [file ijms-20-05548-s001.zip › Figure S3.tif]

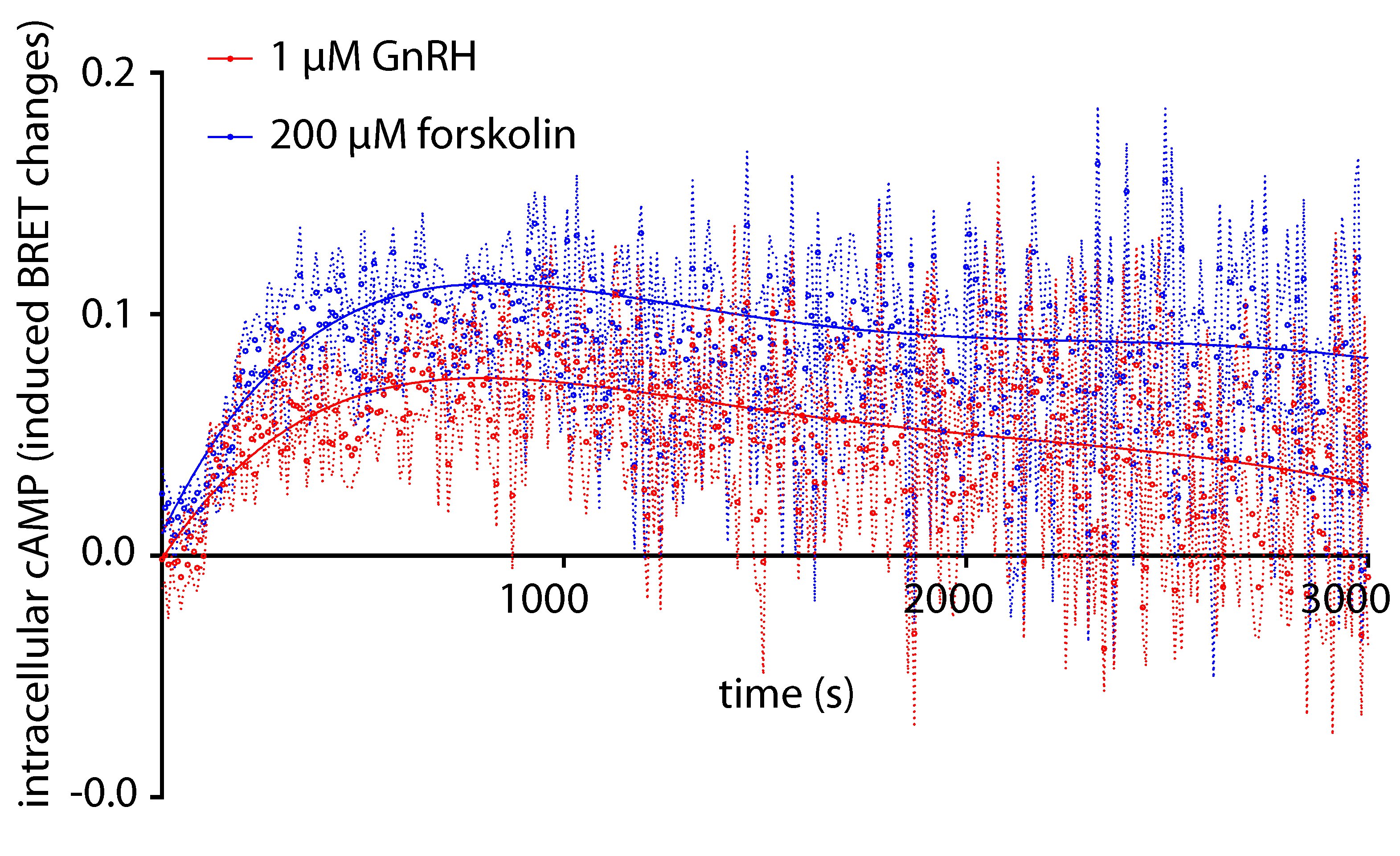

Supplement: Supplementary file 1 [file ijms-20-05548-s001.zip › Figure S4.tif]

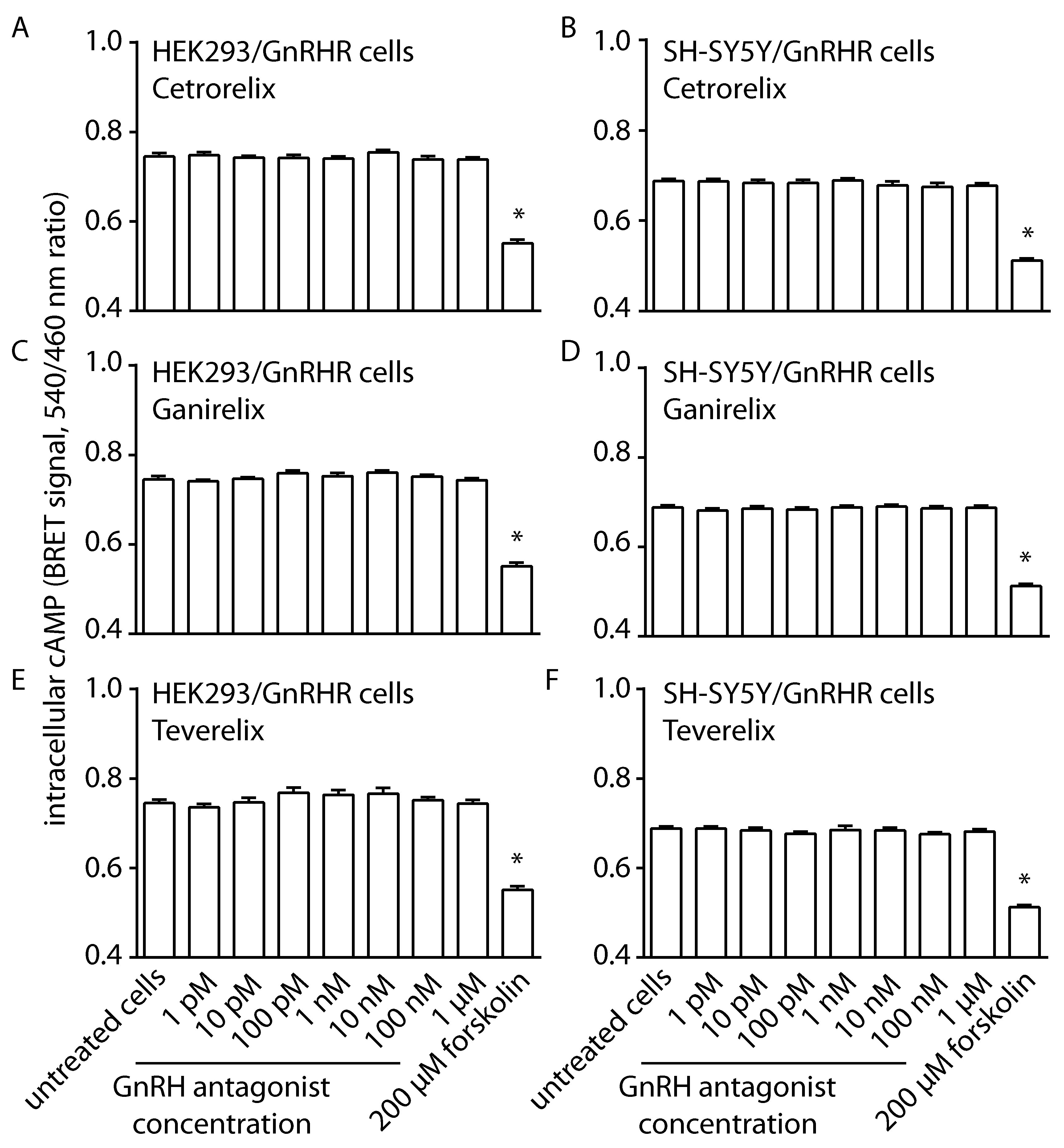

Supplement: Supplementary file 1 [file ijms-20-05548-s001.zip › Figure S5.tif]

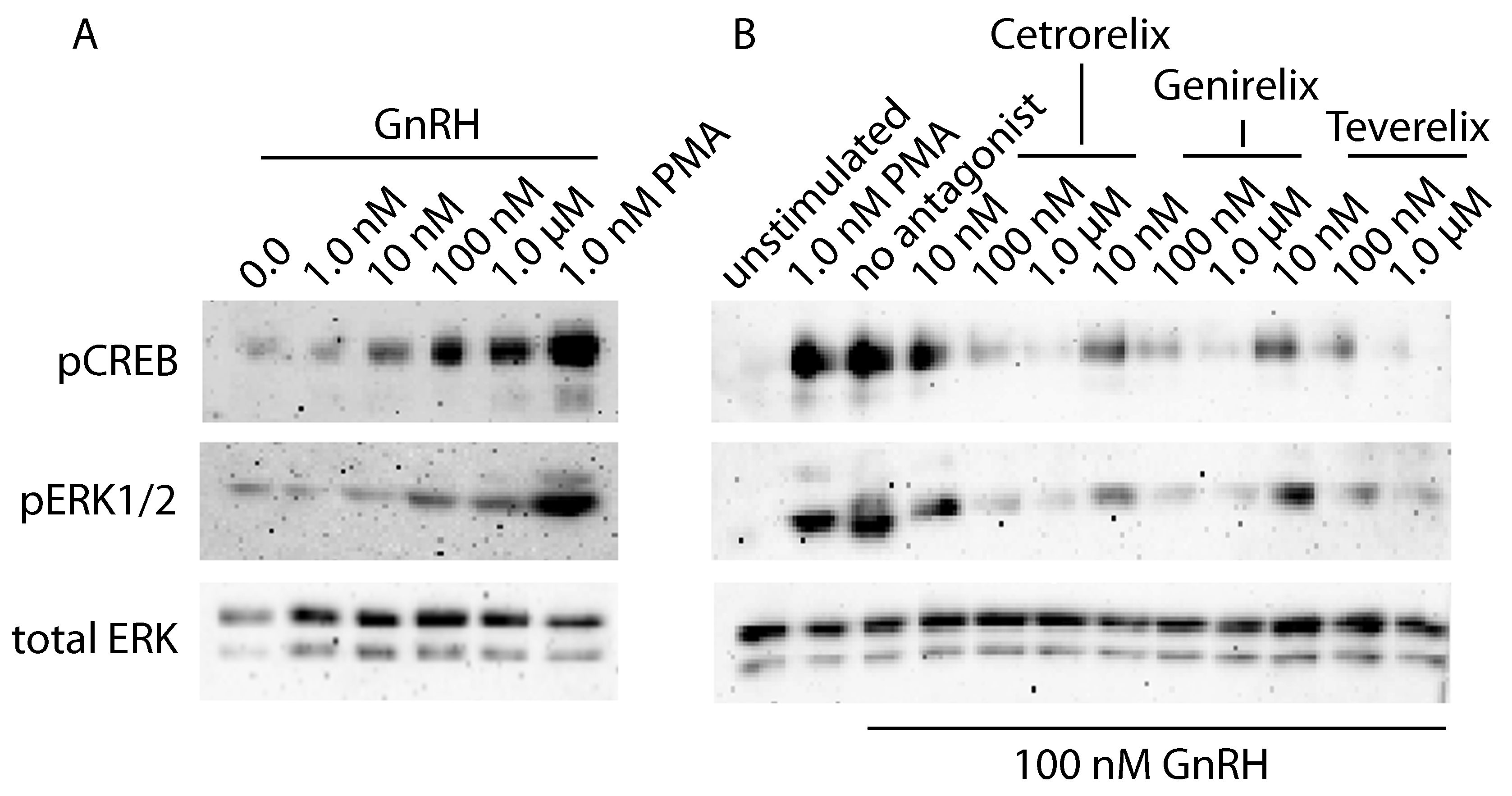

Supplement: Supplementary file 1 [file ijms-20-05548-s001.zip › Figure S6.tif]
